# On the Origin and Progress of Empiricism

**Published:** 1806-04

**Authors:** Duncan Forbes


### On the Origin and Progress of Empiricism. By Duncan Forbes, M. D.

( Continued from Vol. xiv. pp. 437—448. )

In a former paper I have endeavoured to trace the rise and progress of the ancient sect of Empirics; I have also attempted to prove, that this very respectable class of physicians were empirics in the good and proper sense of that term, and that their conduct and practice were diametrically opposite to the conduct and practice of their impudent, illiterate, and irrational successors in modern times, who pay no attention to nature in the cure of diseases, follow unconditionally their own crude undigested ideas, or implicitly adopt those of others.

In the progress of my inquiry, I arrived in my former Essay, at that stage of it when the various tribes of collectors and workmen, whose province it formerly was to bring the simple and compound medicines ready for use, to the *apotheca*, or *repository* kept in the house of the pharmaceutic physician, at length availed themselves of such superficial knowledge of their qualities, as they casually collected, to usurp the office and dignity of their masters.

Thus did the empirics insensibly descend to the level of the vulgar; or rather, through the intervention of the chemists of the latter ages, the sect was new modelled. They now neglected all erudition, and even disregarded the history and symptoms of diseases; the causes of diseases they now explained in their laboratories; and from the result of their pharmaceutical processes, adopted the modes of cure. They arbitrarily assigned certain remedies to particular parts of the body, on which they were supposed to exert their effects by a peculiar predilection. Hence, the classifications of pectorals, of stomachics, of hepatics, of cephalics, of cordials, &c. One medicine attracts and eliminates the bile, another the pituita, and a third the atrabilis or melancholy. Some preparations irradiate radiate the animal spirits, by others they are darkened and obscured.

Such were the idle conceits of the chemists, of which our modern quacks are the servile imitators. Serapion and his followers, while duly attentive to the operation of remedies, assiduously explored the nature of the disease; our modern empirics confine their range within a more limited sphere of observation: To an acquaintance with medicine they attach some degree of importance, but the diseased actions of the system form no part of their concern. The empirics of antient times may therefore comparatively be regarded as true physicians, while the highest rank which justice can assign to their modern brethren is, that of an ignorant conceited apothecary's apprentice.

Folly and sound reason cannot be exhibited in a more contrasted opposition, than in the conduct of the modern quack, when compared with the department of the scientific practitioner. The latter assiduously repairs to those sources of knowledge from which the latter turns with disgust; because every conscientious man, who in his daily practice finds himself involved in perplexity and darkness, would wish to avail himself of the experience of his predecessors. General science and medical literature are constantly regarded by the true physician as the luminaries which must dissipate the gloom which too frequently overhangs him in his progress; but in the estimation of the empiric, these luminaries are stripped of their importance, and to his eye, the light which they afford is less interesting, because the number and the nature of diseases are already determined, and exactly correspond with the evident or occult qualities of his nostrums. To the experience of others our infallible quack seldom attaches any consideration; neither does it merit his regard in what effect a different treatment of a similar disease resulted. In restoring the system to a healthy action, nature, in the empiric's opinion, is indeed a feeble agent; but then, every obstacle must give way to the sovereign efficacy of his specific. It is sufficient that a medicine posesses a certain virtue; and unavailing would be the attempt to imitate nature in the solution of the disease; every thing depends on the remedy; and no dependence can be placed either in the efforts of nature, or in the prudence of the physician.

It has been well observed, that the less we know of the material world, the more extensive we suppose our acquaintance to be with the world of spirits; and there can be no doubt, that from ignorance of the operations of nature, and of the laws of the animal economy, has originated that predilection for superstitious remedies, which in every age has constituted so prominent a feature in the character of the people. The people have neither leisure nor inclination to reason; credulity is of course for them more convenient, than the researches necessary for the investigation of truth. This credulity the medical impostor knows how to turn to his account; it, indeed, accords with his own capacity, for he too is not greatly gifted with a talent for disquisition; and to affix a barbarous name to a universal specific, is a less arduous task than to study the nature of a disease, and to apply its appropriate remedy.

It is an observation of the great Hippocrates, that a physician ought to possess a tranquillity and dignity of mind superior to superstition of every kind; because it is impossible, says he, to be superstitious and at the same time to see the truth. This incomparable man, at a time when physic was entirely founded on superstition, resisted with energy the torrent of ignorance. In his Treatise on Epilepsy, he exposes in a masterly manner the tricks of impostors, who undertook to cure by means of amulets and charms, diseases which they were unable to relieve; and there is not a single instance in his own voluminous writings, which savours of credulity or superstition.

Happily for mankind, in the greater part of Europe, the empire of superstition is greatly circumscribed; but there are principles in human nature, which to a less or a greater degree will perpetuate its domination, and to the most superficial observer it cannot fail to be obvious, that over medicine it exercises a very despotic influence. It is surely no less absurd to determine the nature of a disease from inspecting the contents of the urinal, than from the flight of birds to predict the fate of an empire; and yet there are thousands and tens of thousands, who acquiesce in the urine doctor's decision with a confidence equally implicit as the Roman populace ever received the predictions of the avispex.

Impostors of this cast are not confined to Britain. In Langneau, a village of the *ci-devant* Swiss Cantons, there lived not many years ago, and for aught I know, there may be still in being, a certain *Michael Schuppach*, whose fame, at one period of his career, was so widely diffused, that patients flocked to him from almost all the countries of Europe. He confined himself wholly to the inspection of the urine. During the summer months the village of Langneau

Langneau was constantly crowded with visitants from Basle, Berne, and other towns of Helvetia, some of whom went to consult *Schuppach,* others from a mere impulse of curiosity, to witness the procedures of this rustic physician, who always received his guests in his night cap, and waist-coat without sleeves, for he never wore a coat. He kept an excellent *table d' hote,* and was very moderate in his charges. Many of his patients were of the highest rank, and even diplomatic characters were occasionally found at the levee of this empiric. He erected a handsome building for their reception near his hut, and likewise a laboratory in which he prepared his medicine. A German physician, of reputation and ability, attempted to undeceive the people, by exposing the artifice of *Schuppach,* and published a small treatise upon the subject. The people, however, as is too commonly the case, attributed this well-meant performance more to jealousy than to philanthropy.

The vulgar regard the urine as a mirror in which all the internal operations of the body are immediately perceptible. They therefore expect that the physician, from the bare inspection of this excrementitious fluid, and disregarding every other symptom, should not only ascertain the nature of the disease, but also decide on the patient's constitution. Ignorance naturally produces a predilection for the marvellous; and accordingly, proportionate to their ignorance, do their prejudices take the firmer hold of the minds of the people; and there have been physicians, who from corresponding ignorance, or from knavery, not only sanctioned and patronized these impositions, but indulged themselves in similar reveries.

The period is not very distant when we might observe an urine castor inspect his patient's water with such a mysterious air, as could only be acquired by a habit of the most studied imposition. Enlightened people, however, are now no longer the dupes of these gross deceivers, although they have still their patrons among the credulous and the ignorant. This assertion, however, is not general in its application, as some there are, who, on other occasions, are defective neither in genius nor in talents, that yet, in matters relative to their health, seem to abjure all their knowledge and good sense, and discover an equal degree of credulity with the most ignorant peasant.

A pleasant story to this effect is recorded by Zimmerman, in his Treatise on Experience in Physic. " A man of merit," says he, " after wavering for some time in his opinion concerning a celebrated urine castor, declared at length in his favour, and from that instant praised him in all companies. A waggish student happened to say to him, soon after this, that his new favourite was one of the cleverest fellows in the world, having predicted from the urine of a cat, that there would be no more mice for a year to come. Sorely chagrined at this keen raillery, he returned home, and preparing a mixture of urine, and tincture of safron and chalk, sent it by his servant to the urine doctor, with a note, to inform him that it belonged to a person who had been long sick, and who wished for his advice. The quack," continues Zimmerman, "who has still the reputation of a very skilful physician, receives his fee and sends back his opinion in writing, describing the nature of the disease, and the method of cure. The good man now saw and acknowledged the imposition; and surely, his good sense ought to have led him to this before."

The world has always been duped with absurdities of this kind. The following story will be found more amply detailed in M. Desault's *Dissertation sur la Goutte*. To many of the readers of the Medical Journal, it may appear to exceed the bounds of belief; Desault is, however, an suthor of credit, and similar marks of folly and superstition every day meet our observation.

About the year 1698, an illiterate peasant, named *Christopher Ozanne*, became so famous for his skill in physic, that coaches regularly set off three times a week from Paris, filled with patients, who went to consult *Ozanne*, at Chandray, in Normandy, at the distance of forty leagues. The patient's names were all inserted in a book as fast as they arrived, and were admitted by turns to an audience, without any distinction of rank. *Ozanne* received them in a smoaky hut, and his prescriptions were usually of some simples. He delivered his opinion in a very blunt manner, and admitted of no expostulation or reply. This singularity of behaviour proved at length the cause of his fall. A young lady, who had been married only three months, went to consult him, and related all her complaints. The husband, who accompanied her, added every circumstance which he thought could throw light on the nature of his wife's disorder. When they had finished the history of the case, "Madam," said *Ozanne*, "Your complaints are the effects of a lying-in?" It was to no purpose that the young couple remonstrated against the absurdity of such a decision. *Ozanne* shewed them to the door, and called out for more patients.

Daily

Daily experience evinces the danger of a precipitate exhibition of the same remedy in similar diseases; and the following instances, which might be easily multiplied, shew the fatal effects of such an empirical practice. A person violently afflicted with a colic, swallowed a glass of ardent spirits, from which he experienced almost instantaneous relief, as his disease originated from flatulency. Another person, suddenly attacked with similar pains, and induced by its success in the case of his friend, had immediate recourse to the same expedient. He too swallowed a glass of brandy without hesitation, which in a few hours put a period to his existence. In this case the fatal consequence of ardent spirits would, to a person conversant with diseases, be abundantly obvious, as the disease was not really a colic but an inflammation of the intestines. A third person was seized with an excruciating colic, owing to some poisonous mushrooms, of which he had inadvertently *eaten*. The immediate application of an emetic, followed by the exhibition of some diluted vegetable acid, removed the complaint and restored him to health. A fourth had an attack of the same disease, from an encysted collection of matter pressing upon the intestines; but in this case the remedy which was exhibited with success in the former instance, proved necessarily fatal; for it burst the sac, poured out its contents into the cavity of the abdomen, and thus speedily terminated the patient's existence. Another had accidentally swallowed some arsenic, which occasioned excruciating pains of his bowels, resembling those of a common colic; large doses of sweet oil were immediately prescribed, which fortunately effected his recovery; but any, or all of the remedies employed in the above cases, would have proved completely inert and unavailing. But let me here close a narrative, which in every human mind, cannot fail to excite the most painful sensations. The subject, however, is abundantly copious, and it were easy to extend the details, by bringing forward a long catalogue of fatal but interesting accidents. But to lengthen the illustration would be transgressing the limits within which articles of this nature must necessarily be confined in the Medical Journal.

There is another consideration which merits great attention, although to such as are unacquainted with the laws of the animal economy, its force may not be immediately obvious. If we wish to make any remarkble change in the system of an organized body, we must employ such instruments as bid fair to accomplish our end without too violent an impression upon the vital principle; in other words, without extending their action beyond a limited length. It is not indeed to be denied, that by a gradual indulgence, a person may be habituated even to the strongest stimulants; but, it is equally certain, that this habit is acquired at the expence of palsied organs, and of a broken constitution; such are the deplorable consequences of imposture and of credulity! Were it possible to enumerate all the victims of this mysterious infatuation, the swelled catalogue would, probably, prove it a more potent engine of destruction, than those desolating agents, gunpowder and the sword.

On this subject, it has been well remarked, "that in the present order of things, it is easier to cheat a man out of his life than of a shilling, and almost impossible to detect or punish the offender. Notwithstanding this, people still shut their eyes, and take every thing upon trust that is administered by any pretender to medicine, without venturing to ask him a reason for any part of his conduct. Implicit faith, every where else the object of ridicule, is still sacred here." Thus it is, that illiterate and audacious empirics sport with the lives of a credulous public, who seem obstinately bent on shutting their ears against all the dictates of reason and experience; and thus is it, that the fund of public credulity proves an inexhaustible source for all such as resolve to levy contributions on it. In vain is the spirit of quackery exorcised in one form; it rises again immediately, "*with twenty ghastly murders on its head to push us from our stools.*"

The progress which intellectual philosophy has made in the last century, is justly a subject of exultation; but as to the point in question, I know not what advantages reason has gained over the darkest periods of the middle ages. We boast that we live in enlightened times, yet do our great cities teem with hosts of Quacks and Mountebanks, who by the exhibition of their tinctures, and essences, and balsams of life, have ingratiated themselves even with the most polished classes of the citizens. Do we not daily hear of *celestial beds?* and are not our understandings insulted by a pompous display of the enchanting powers of *animal magnetism?* Are not our delicate, credulous females persuaded to wear *achromatic belts*, pervaded by an universal magnetic spirit, whose sovereign virtues can heal every disease incident to the human frame, even broken limbs and exfoliation of bones? Need I notice the many opulent votaries of *Perkinism*, who who by a strange infatuation are persuaded to purchase a pair of metallic skewers, termed *tractors*, not worth a sixpence, at the enormous* price of five guineas? or advert to the table of blood-letting, and other absurdities which still find a place in popular almanacs, to prove that the influence of Charlatanism is widely diffused; and that even our age is far from being the "age of reason," as it has been exultingly termed? We can contemplate the progress of real knowledge with pleasure; but we observe with regret, that the temple of superstition is still crouded with numberless votaries; that human reason is still the slave of the most tyrannical prejudices; and that the most successful way of exciting general attention is to affect the mysterious and the marvellous. "Medical superstition," says a celebrated physician on the continent, "is to be sought not only in the peasant's hut, but in the city, in the palace beside the toilette of the lady of the first fashion, and in the cabinet of the philosopher; more or less disguised, perhaps, but still the same in substance."

Treatises on Domestic Medicine, and other popular works of this kind, have greatly increased the mischief, without diffusing one solitary ray of information, for medicine cannot be learned from books, much less from a single volume, which professes to cure the long list of diseases "to which mortality is heir." This source of evil is probably irremediable; for popularity has charms. Men from various motives, are fond of commencing authors; and happily the freedom of the press, although teeming with mischief, still continues.

Mankind are fond of mystery; and it is more congenial with a sick man's mind to expect relief from the occult qualities of a medicine, than from its sensible virtues. Hence, in a great measure, arises the success of many boasted secret remedies, which, when compounded in an apothecary's shop, instantly lose their efficacy.

Bone setters are another set of empirics, they are real pests to society, and their ignorance can be only equalled by their daring impudence. Of their rough and improper modes of practice, our hospitals shew melancholy instances.

If consistent with rational freedom, the interference of the Legislature appears in no instances more requisite than in suppressing these noxious tribes, against which I have, in the preceding Essay, pointed my indignant, well-meant rebuke. Patent medicines in particular, are a disgrace to our Government: They impose upon the vulgar, who imagine that the wisdom of the Legislature has thought it it proper to reward the inventor by a public mark of favour. This, in my opinion, is a subject which merits the attention of our Colleges. They, as guardians of the public health, should consider it as a duty incumbent upon them, to expose the dangerous errors of these people; and even to remonstrate against the facility with which their nostrums get into general circulation, under the sanction of a Royal Patent.

But while we speak with just contempt of those men who *chouse* a credulous public with their Botanical Syrups, and their Cordial Balsams, why do we pass over in silence those who apostatize from amongst ourselves? Ought not they to have some mark of contempt, or, at least, of disapprobation attached to them? The Chinese apostate *Chingse*, so celebrated of late for his brown and yellow lozenges, vends at an enormous price jallap and calomel, which country practitioners, on both sides of the Tweed, use, and have used, as anthelmentics before he was born; and yet he calls himself a regular practitioner.*

*Edinburgh, January* 4, 1805

* "A foolish idle fellow at Florence, hearing that a physician had obtained great credit and wealth by the sale of some pills, undertook to make pills himself. He administered the same pills to all patients whatsoever; and as, by chance, they sometimes succeeded, his name became famous. A countryman called on him, and desired to know if his pills would enable him to find an ass he had lately lost. The quack bid him swallow six pills. In his way, the operation of the pills obliged him to retire into a wood, where he found his ass. The clown spread a report, that he knew a Doctor who sold pills, which would recover strayed cattle." See Spirit of French Anas, lately published, Vol. i. Article PAGGRANA.

CRITICAL

